# Genetic and developmental analysis of differences in eye and face morphology between *Drosophila simulans* and *Drosophila mauritiana*

**DOI:** 10.1111/ede.12027

**Published:** 2013-05-16

**Authors:** Saad Arif, Maarten Hilbrant, Corinna Hopfen, Isabel Almudi, Maria D S Nunes, Nico Posnien, Linta Kuncheria, Kentaro Tanaka, Philipp Mitteroecker, Christian Schlötterer, Alistair P McGregor

**Affiliations:** a Department of Biological and Medical Sciences, Oxford Brookes UniversityGipsy Lane, Oxford OX3 0BP, United Kingdom; b Institut für Populationsgenetik, Veterinärmedizinische Universität WienVeterinärplatz 1, A-1210 Vienna, Austria; c Department of Population Genetics, National Institute of GeneticsMishima, Shizuoka 411-8540, Japan; d Department of Theoretical Biology, University of ViennaAlthanstrasse 14, A-1090 Vienna, Austria

## Abstract

Eye and head morphology vary considerably among insects and even between closely related species of *Drosophila*. Species of the *D. melanogaster* subgroup, and other *Drosophila* species, exhibit a negative correlation between eye size and face width (FW); for example, *D. mauritiana* generally has bigger eyes composed of larger ommatidia and conversely a narrower face than its sibling species. To better understand the evolution of eye and head morphology, we investigated the genetic and developmental basis of differences in eye size and FW between male *D. mauritiana* and *D. simulans*. QTL mapping of eye size and FW showed that the major loci responsible for the interspecific variation in these traits are localized to different genomic regions. Introgression of the largest effect QTL underlying the difference in eye size resulted in flies with larger eyes but no significant difference in FW. Moreover, introgression of a QTL region on the third chromosome that contributes to the FW difference between these species affected FW, but not eye size. We also observed that this difference in FW is detectable earlier in the development of the eye-antennal disc than the difference in the size of the retinal field. Our results suggest that different loci that act at different developmental stages underlie changes in eye size and FW. Therefore, while there is a negative correlation between these traits in *Drosophila*, we show genetically that they also have the potential to evolve independently and this may help to explain the evolution of these traits in other insects.

## INTRODUCTION

Understanding the evolution of morphology remains a central goal of evolutionary biology. This requires the identification of (1) the causative genomic regions and genetic changes responsible for morphological differences, (2) how these changes alter developmental programs, and (3) the underlying evolutionary forces. While great progress has been made in understanding the precise genetic and developmental basis of variation in some traits, including trichome, bristle, and pigmentation patterns in *Drosophila*, and pelvic armor in sticklebacks (e.g., Hoekstra et al. 2006; McGregor et al. 2007; Rebeiz et al. 2009; Chan et al. 2010), less is known about the changes underlying the evolution of differences in organ shape and size (True et al. 1997; Pavlicev et al. 2008; Matta and Bitner-Mathe 2010). This is in part because organ shape and size are often composites of multiple traits with coordinated development, which can potentially influence each other during development and even evolution (Lande 1980; Smith et al. 1985; Brakefield 2006). For example, the overall proportions of the *Drosophila* head are determined by the relative size and shape of the compound eyes, head cuticle, ocelli, antennae, and maxillary palps, all of which develop from the eye-antennal imaginal discs (Held 2002). Thus, the size of the eyes, for example, will influence the overall width of the head, and because eye size is determined by the number, size, and shape of ommatidia, variation in eye size can potentially be caused by changes in a number of developmental programs and many loci across the genome.

In *Drosophila*, subdivision of the eye-antennal disc begins during the second larval instar in part through antagonism between *wg* and *dpp* signaling. Early retinal genes are activated by Dpp signaling in the posterior of the disc, while in the anterior retinal fate is repressed by Wg through the activation of *homothorax* (*hth*). It has been shown that loss of *wg* expression results in the replacement of head cuticle with retinal tissue, and conversely ectopic Wg signaling causes an increase in head cuticle at the expense of retinal tissue (Treisman and Rubin 1995; Royet and Finkelstein 1996; Lee and Treisman 2001; Baonza and Freeman 2002). Indeed mutations in many of the regulatory factors required for the development and differentiation of the eye-antennal disc can affect the relative proportions of different parts of the head without changing the overall size of the head (Royet and Finkelstein 1996; Amin et al. 1999; Amin and Finkelstein 2000; Thomas and Ingham 2003). However, it is also known that differences in the size of the eye can develop independently of the head cuticle and vice versa (reviewed in Dominguez and Casares 2005; Amore and Casares 2010). For example, in *unpaired* (*upd*) mutants, proliferation of cells in the presumptive retinal field is repressed, which reduces eye growth and gives rise to eyes with fewer ommatidia (Chao et al. 2004). Similarly, mutations in the Myc transcription factors also lead to changes in eye size, but in this case through the regulation of ommatidia size (Steiger et al. 2008).

How does the relationship between the relative proportions of head capsule tissues on one hand and their independent growth on the other, as revealed by using gene knockout or over-expression experiments in *Drosophila*, reflect natural variation in these traits? We and others have described extensive differences in ommatidia number and size, and face width (FW) among species of the *D. melanogaster* species complex (*D. melanogaster*, *D. simulans*, *D. mauritiana*, and *D. sechellia*) (Hammerle and Ferrus 2003; Posnien et al. 2012), and in other *Drosophila* species (Norry et al. 2000). We confirmed that *D. mauritiana* generally has larger eyes than *D. melanogaster* and *D. simulans*, which is caused mainly by a difference in ommatidia size. Moreover, we found that there is a pervasive negative correlation between the face and eye size among all species surveyed in this *Drosophila* complex. Interestingly, the ratio of eye to face is particularly exaggerated among males within and between *Drosophila* species (Posnien et al. 2012). This effect is even more obvious in the onion maggot fly, *Delia antiqua*, where males have larger eyes than females but proportionally smaller faces, and consequently the overall size of the head is similar between the sexes (Dominguez and Casares 2005). Such observations suggest that the evolution of eye and face size may be constrained by their developmental integration in *Drosophila* and other dipterans. Quantitative genetic models predict that functional and developmental integration of traits consequently leads to evolutionary integration, and therefore, their co-evolution (Cheverud 1996). However, in the absence of functional integration, even developmentally related traits (i.e., traits that develop from the same progenitor cell field or undifferentiated tissue) may be regulated by different genetic loci and can evolve independently of one another (Wagner 1996).

To investigate the genetic and developmental basis of differences in eye and face morphology between *D. mauritiana* and *D. simulans* males, we carried out QTL mapping, generated introgression lines, and compared the development of the face and eye fields within the eye-antennal discs of these species. We found that the genomic regions underlying differences in eye and face size are non-overlapping, which suggests that in this case different loci are responsible for making eyes larger and the face cuticle smaller and vice versa. The results of our QTL mapping approach were confirmed by two independent introgressions from *D. mauritiana* into *D. simulans*, whereupon we generated flies with larger eyes but that had no significant difference in FW, and flies with narrower faces but no significant difference in eye size, respectively. Consistent with the genetic distinctiveness of these two traits, we found that differences in eye and face size between these species arise at different stages of development. Taken together, our results show that interspecific differences in the proportions of the head capsule are determined by multiple loci that can act independently on the different tissues at different developmental stages.

## MATERIALS AND METHODS

### Fly strains

*D. simulans yellow* (*y*), *vermillion* (*v*), *forked* (*f*) (hereafter YVF) was obtained from the *Drosophila* Species Stock Center, San Diego, California (Stock no.14021–0251.146). *D. mauritiana* TAM16 is a wild-type, inbred strain, bred from a single female. *D. mauritiana* D1 and Q1 (True et al. 1996) were kindly provided by JP Masly. *D. mauritiana w*^*−*^ and *D. simulans w*^*501*^ were a gift from M. Ramos-Womack. Flies were maintained on a standard cornmeal diet (supplied by Drosophila Lab Services) at 25°C under a 12h:12h dark/light cycle. Eye area and FW were compared between males of the parental strains using a two-sample *t*-test.

### Genetic markers

Genotyping was performed with a mix of visible and molecular markers (Table S1). *D. simulans* YVF carries three recessive visible mutations on the X chromosome (*y*, *v*, and *f*). Fourteen microsatellite and 24 *Eco*RI restriction site differences were identified in the *D. simulans* YVF and *D. mauritiana* TAM16 genome sequences using CLC Sequence Viewer (CLC bio) and Geneious 5.5 (Biomatters Ltd) (Table S1). DNA was extracted from whole flies using an adapted salt extraction protocol. Microsatellite genotyping was performed using a MegaBACE 500 Sequencer (GE Healthcare) and Genetic Profiler v.2.0 (GE Healthcare), and regions with restriction site differences were PCR amplified, digested, and analyzed on agarose gels.

### Phenotypic measurements

Eye area measurements were taken from frontal images of the head, as described in Posnien et al. (2012), and the width of the cuticle between the eyes (FW) was measured at the height of the orbital bristles just above the antennae (Fig. S1). These frontal images were annotated with landmarks as described in Posnien et al. (2012). The length of the T1 tibia was measured to account for body size. We lacked tibia measurements for 14 individuals in the backcross population used for the QTL mapping, and therefore, used the population mean for these flies. Ommatidia number and facet diameter were determined from Scanning Electron Micrographs as described in Posnien et al. (2012).

### Backcross design and statistical analyses

Virgin *D. simulans* YVF females were mated to *D. mauritiana* TAM16 males. F1 virgin females were then backcrossed to *D. mauritiana* TAM16 males. A total of 244 males from the backcross progeny were genotyped and phenotyped. QTL analyses were performed in R/qtl (Broman et al. 2003; R Development Core Team 2012). The genetic map was constructed using default parameters and the Kosambi map function with a total of 34 markers across the 2nd, 3rd, and X chromosomes. To determine QTL locations we performed QTL scans using several different methods including: standard interval mapping with Haley-Knot regression (Haley and Knott 1992), composite interval mapping (CIM) (Zeng 1994), and a native R/qtl algorithm (*stepwiseqtl*) (Broman and Sen 2009) that employs a QTL model search allowing for multiple linked QTL and interactions among all QTL. Genome-wide statistical significance thresholds (1%) were determined for each phenotype (eye area and FW) using 1000 permutations. For CIM analysis we used an arbitrary threshold of LOD = 3, which is slightly more conservative based on significance thresholds calculated for other analysis. We calculated 2-LOD support intervals for all significant QTL, and tested for any possible epistatic interactions between significant QTL by fitting full linear models in an ANOVA framework (*F*-tests, type III sum of squares) with all significant QTL as fixed effects and all pair-wise interactions between them. Furthermore, we estimated additive allelic effects of all significant QTL in three ways (as listed in Table 2): (1) the difference in phenotype averages between homozygotes (*D.mau*/*D.mau*) and heterozygotes (*D.mau*/*D.sim*) for autosomes and hemizygotes for the X chromosome (effect size), (2) differences in (1) standardized by half the difference between parental lines for autosomes and the entire difference in the case of the X chromosome (relative effect size), (3) the percentage of phenotypic variation accounted for by the significant QTL in the backcross population (variance explained).

### Introgression lines

We introgressed the region between *y* and *v*, from *D. mauritiana* TAM16 into *D. simulans* YVF in at least three replicate lines (X introgression, Fig. S2). For this, *y*^*+*^*v*^*+*^*f*^*−*^ virgin females were selected from the progeny of a F1 × *D. simulans* YVF backcross, followed by repeated generations of backcrossing of *y*^*+*^*v*^*+*^*f*^*−*^ virgin females to *D. simulans* YVF males. Five replicates were set up per line, per generation. At the end of the egg-laying cycle of each generation, mothers were collected and genotyped for four molecular markers equidistantly spaced between *y* and *v* (Table S1). The next generation was then setup with a single replicate using virgin *y*^*+*^*v*^*+*^*f*^*−*^ daughters from a female with no double recombination events in the introgressed region. At backcross generation 7, we sampled sibling males, across three different replicates and measured eye area, FW, and tibia length (TL), as well as placing landmarks on frontal images of the head (Fig. S1). The same introgression procedure was repeated to obtain *y*^*+*^*v*^*+*^*f*^*−*^ and *y*^*−*^*v*^*−*^*f*^*−*^ individuals for comparison of ommatidia facet diameter and ommatidia number. The experimental setup was identical to the first introgression except this time we sampled *y*^*+*^*v*^*+*^*f*^*−*^ (*n* = 20) and *y*^*−*^*v*^*−*^*f*^*−*^ (*n* = 20) males at backcross generation 5 pooled from two replicate lines.

The region between the markers D1 and Q1 (True et al. 1996) (Table S1) was introgressed from *D. mauritiana w*^*−*^ into *D. simulans w*^*501*^ (3L introgression, Fig. S2). For this, the F1 of a cross between D1 and Q1 lines was crossed to *D. mauritiana w*^*−*^, to generate heterozygous *D1*^*+*^*Q1*^*+*^ flies. These were backcrossed to *D. simulans w*^*501*^ using a similar strategy to that described for the X chromosome, using four genetic markers to select against double recombination events (Table S1). After four generations, males from three replicate lines were collected for FW and eye area measurements. All statistical comparisons were made with a two-sample *t*-test after an *F*-test for equality of variances.

### Morphometric analysis

The landmark configurations of flies from the X chromosome introgression (carried out for seven generations), along with males of the parental lines (TAM16 and YVF) were analyzed by landmark-based geometric morphometrics (GM) (Rohlf and Slice 1990; Mitteroecker and Gunz 2009). GM approaches generate shape variables from a set of anatomically homologous *x, y* coordinates after eliminating variation due to differences in orientation, position and scale. A total of 15 fixed (white circles, Fig. S1B) and 30 semi-landmarks (gray circles, Fig. S1B) were digitized on frontal images of dissected heads (for further details on treatment of semi-landmarks see Posnien et al. 2012) using the tpsDIG2 software (Rohlf 2010). A generalized procrustes analyses (Rohlf and Slice 1990) was performed on the entire set of landmark coordinates to remove variation arising from differences in position, orientation and size. We analyzed the pattern of individual shape differences by a principal component analysis (PCA) of the adjusted shape coordinates and visualized the PC axes by thin-plate spline (TPS) deformation grids. The TPS deformation grids represent deviations from the average landmark configuration of the entire dataset. We found that the first two principal components of the resulting shape coordinates reflect variation in the orientation of the head during the mounting process. We removed these effects by projecting the data into the subspace perpendicular to these two principal components. In addition, the resulting landmark configurations were symmetrized by averaging each configuration with its relabeled reflection (Mardia et al. 2000). All PCA analyses and TPS deformation grids presented were based on this final set of adjusted coordinates. All morphometric analyses were carried out in Mathematica 8.0.

### Immunostaining of eye-antennal imaginal discs

Flies for dissections were raised in density-controlled conditions by starting each vial with 30, freshly hatched, LI larvae. LIII larvae were collected at 120 h after egg deposition and males were selected on the basis of the morphology of their genital discs. Immunostaining with mouse anti-Eya antibody (Developmental Studies Hybridoma bank) was performed at 1:100 dilution using standard protocols, followed by anti-mouse-Cy3 (Jackson Immuno Research) secondary AB staining, at 1:200. The actin cytoskeleton was stained with Alexafluor 488-Phalloidin (Molecular Probes) at 1:40 dilution for 30 min after discs fixation. Discs were mounted in Prolong Gold antifade reagent, supplemented with DAPI (Molecular Probes), and captured with a Zeiss LSM 510 confocal microscope. Images were processed using NIH ImageJ software. Areas from eye and face fields were measured using Adobe Photoshop CS5.

## RESULTS

### An eye size QTL of large effect maps to the X chromosome

**Figure 1:**
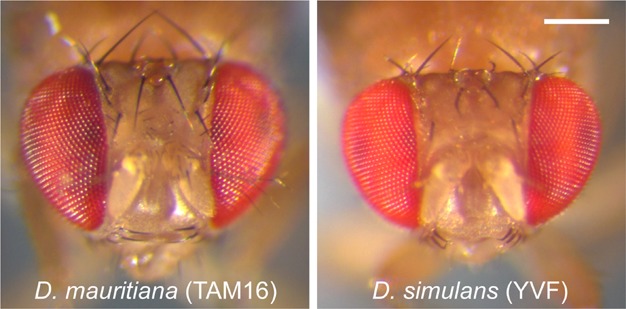
Heads of parental strains. Frontal images of heads of the *D. mauritiana* strain TAM16 and the *D. simulans* strain YVF show that the eyes of TAM16 are larger, but that the cuticle between the eyes of this strain (i.e., the face) is narrower. Both images were taken of male flies, at the same magnification. Scale bar = 200µm.

**Figure 2:**
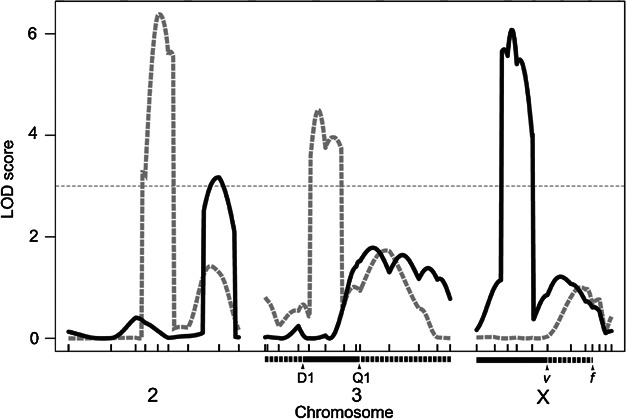
QTL affecting eye area and face width. LOD profiles from a CIM analysis of eye area (solid-black curves) and face width (gray-dashed curves), both adjusted for body size using residuals from regression on tibia length. The horizontal-dashed line represents a threshold of LOD = 3. Ticks on the *x*-axis represent genotyped markers spaced in cM across chromosomes 2, 3, and X. Black bars underneath chromosomes X and 3 indicate the regions introgressed from *D. mauritiana* into a *D. simulans* background (X and 3L introgression lines, see text). Differences in recombination breakpoints between *v* and *f* among lines in the X introgression, and outside D1 and Q1 in the 3L introgression are indicated by the dashed parts of the bars underneath chromosomes X and 3.

*D. mauritiana* has significantly larger eyes than *D. simulans* (Fig. 1; Table 1, *t* = 13.13, df = 24, *P* < 0.001) (Posnien et al. 2012). To map the genomic regions responsible for this morphological difference, we used a QTL backcross mapping approach, between the *D. simulans* YVF and *D. mauritiana* TAM16 strains. Using CIM for eye area values (EA) we found two peaks with significant LOD score peaks on the X chromosome at 34 and 93 cM (Fig. S3; Table 2). We did not identify any significant QTL on either chromosome 2 or 3 (Fig. S3; Table 2). Because the peak for EA at X:93 cM coincided with a peak for TL (Fig. S3), and generally eye size is influenced by overall body size, we repeated CIM using the residuals of EA regressed on TL (EA^TL^). This adjustment resulted in a loss of the peak at X:93 cM, while only slightly shifting the largest peak, to X:33 cM. It also revealed a second, less significant, peak at 142 cM on chromosome 2 (Fig. 2; Table 2).

**Table 1:** Head proportions of males from the parental strains

Species (strain)	EA (µm^2^)	*N*	SD (µm^2^)	FW (µm)	*N*	SD (µm)
*D. simulans* (YVF)	190,310	27	6893	357.4	52	7.1
*D. mauritiana* (TAM16)	218,460	20	7770	342.9	25	8.5

EA, eye area, based on Posnien et al. (2012); FW, face width.

**Table 2:** QTL for eye area, face width, and tibia length

			2 LOD support region (cM) 1		2 LOD support region (mB) 1		Additive allelic effects 2		
Trait	Peak location (Chr: cM)	Peak significance (LOD)	From	To	From	To	Effect size (µm (SE))	Relative effect size (%)	Variance explained (%)
EA^TL^	X: 33	6.07	23	52	X: 2.6	X: ∼8	−6239 (1179)	44.1	10.0
EA^TL^	2R: 142	3.17	127	158	2R: 9.8	2R: 16.1	−4557 (1124)	64.3	3.5
EA	X: 34	5.69	23	52	X: 2.6	X: ∼8	−7062 (1668)	25.1	6.7
EA	X: 93	3.52	70	120	X: 11.7	X: 15.3	−7183 (1735)	25.5	6.1
FW^TL^	2L: 86	6.32	75	100	2L: 14.2	2R: 4.4	−9.21(1.62)	149.1	11.2
FW^TL^	3L: 50	4.68	42	73	3L: 8.5	3L: 16.1	7.52 (1.72)	121.7	7.8
FW	3L: 46	6.19	26	57	3L: 6.2	3L: 12.1	10.00 (2.07)	137.9	8.8
FW	X: 95	4.24	88	119	X: 11.7	X: 15.3	−9.76 (2.14)	68.9	8.1
FW	3L: 126	2.96	75	158	3L: 16.1	3R: 4.5	9.17 (2.3)	126.4	5.8
TL	2L: 72	3.56	57	88	2L: 12.4	2R: 4.0	7.88 (1.92)	252.5	7.5

1 Regions corresponding to the 2-LOD support interval based on the next marker position closest to the interval boundaries.

2 See main text (Materials and Methods section) for details on how these different measures of effect size were calculated.

As an alternative method of analysis, we then used the native R/qtl *stepwiseqtl* algorithm (Broman and Sen 2009), and found highest support for a single QTL model (data not shown). Accordingly, we performed single QTL scans for EA with, as covariates, either TL alone, or both TL and the highest peak (X:93 cM). The results were very similar to our EA^TL^ CIM analysis, and indicated a region of high significance on the X chromosome with a large peak on the X chromosome and a second peak on 2R (Fig. S4A).

The directional effects of the two EA^TL^ QTL were consistent with the eye area differences between these species, that is, flies that were heterozygous at these regions exhibited reduced eye area compared to flies that were homozygous for *D. mauritiana* alleles (negative effect estimates values, see Table 2). The two QTL accounted for about 44% and 64% respectively, of the eye area differences between the species, and explained 10.0% and 3.5% of the variation in the mapping population (Fig. 1; Table 2). No epistatic interactions between the two EA^TL^ QTL were found (*F* = 0.34, df = 1, NS).

### QTL for FW and eye size map to different regions of the genome

*D. mauritiana* has evolved a narrower face compared to *D. simulans* (Fig. 1; Table 1, *t* = 7.89, df = 75, *P* < 0.001) (Posnien et al. 2012). We therefore investigated whether the QTL for eye size and FW overlapped.

A CIM map for uncorrected FW revealed two QTL on the 3rd chromosome, with peak LOD scores at 46 and 126 cM, and one on the X chromosome, with the highest LOD score at 95 cM (Fig. S3; Table 2). Because the latter peak coincided with a QTL for TL, we also repeated this analysis using the residuals of FW regressed on TL (FW^TL^, Fig. 2; Table 2). This adjustment diminished support for both QTL on chromosome 3, though the peak on 3L remained significant. Moreover, as for EA, adjusting for TL resulted in a loss of the X:94 cM peak, suggesting that FW is also influenced by total body size. Furthermore, a significant QTL for FW^TL^ appeared on 2L, at 86 cM, which is not present in our FW analysis (Fig. 2; Table 2). Single QTL scans, performed as described for EA^TL^, gave results that were similar to our FW^TL^ CIM analysis (Fig S4B). The results of both analyses thus show that neither of the FW^TL^ QTL overlapped with any of the EA^TL^ QTL (Table 2). Another difference between our FW^TL^ and EA^TL^ results is evident in the allelic effects of the identified QTL. Whereas both EA^TL^ QTL have moderate effect sizes consistent with the direction of the eye area difference between the parental lines, the two FW^TL^ QTL (Table 2) appear to have much larger effect sizes. Moreover, the direction of the effects of the latter QTL are in opposite directions, with QTL 3L:50 cM in the same direction as the difference between the parental species (Table 2).

In summary, we did not find any QTL for FW that overlapped with any of the QTL for eye area, suggesting that the most significant loci underlying variation in these traits between *D. simulans* and *D. mauritiana* are different.

### Separate introgressions of QTL regions on chromosomes X and 3L confirm that eye area and FW are genetically separable traits

**Figure 3:**
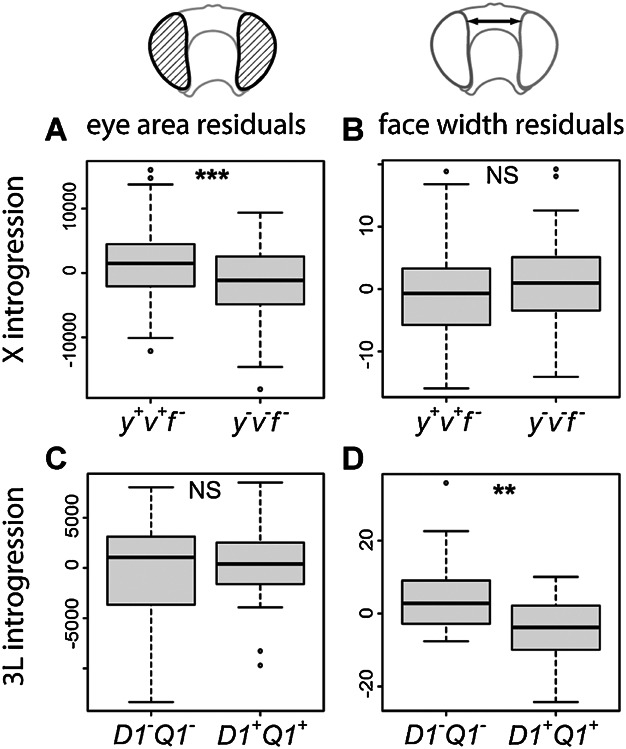
Eye area and face width in the introgression lines. Box plots illustrate residuals of a linear regression of eye area (A, C) and face width (B, D) on T1 tibia length to account for variation in body size. (A–B). X introgression lines at backcross generation 7, comparing eye area residuals (A) and face width residuals (B) in males that are hemizygous for the EA^TL^ QTL from *D. mauritiana* (TAM16) in a *D. simulans* (YVF) background (*y^+^v^+^f^−^*, *n* = 106, EA residuals (mean ± SE) = 1518.61 ± 538.86, FW residuals = −0.65 ± 0.67) to sibling males without the *D. mauritiana* QTL allele (*y^−^v^−^f^−^*, *n* = 109, −1533.36 ± 531.99, 0.66 ± 0.61). (C–D) 3L introgression lines at backcross generation 4, comparing eye area residuals (C) and face width residuals (D) in males that are heterozygous for the FW^TL^ QTL from *D. mauritiana* (*w^−^*) into a *D. simulans* (*w^501^*) background (*D1^+^Q1^+^*, *n* = 24, EA residuals (mean ± SE) = 129.68 ± 828.74, FW residuals = −4.75 ± 1.90), compared to sibling males that are homozygous for the *D. simulans* QTL allele (*D1^−^Q1^−^*, *n* = 24, −129.68 ± 988.80, 4.74 ± 2.13).

In order to verify our QTL results, we carried out two independent introgressions of chromosomal regions underlying major QTL for eye area and FW, respectively. First, we introgressed the X-chromosomal region between *y* and *v* (approximately 8.3 Mb) from *D. mauritiana* (TAM16) into *D. simulans* (YVF) (X introgression) (Fig. 2) After seven generations of backcrossing (BC7), we observed that flies with the introgressed region (*y*^*+*^*v*^*+*^*f*^*−*^) had significantly larger eye areas than recombinant flies from the same lines that had lost the region (*y*^*−*^*v*^*−*^*f*^*−*^) (*t* = 3.95, df = 205, *P* < 0.001 Fig. 3A). This is consistent with the direction of the EA^TL^ QTL that we detected in this region (Table 2; Fig. 2). Moreover, flies either with or without the introgressed region did not differ with respect to FW (Fig. 3B, *t* = 1.49, df = 205, NS).

We also introgressed the QTL region on chromosome 3L, which contributes to variation in FW, but not eye area (Table 2; Fig. 2). To follow this introgression, we performed this experiment using a white-eyed *D. mauritiana* strain carrying the visible markers D1 and Q1 that flank the QTL region and are separated by about 14–15 Mb (True et al. 1996) and *D. simulans w*^*501*^ (Posnien et al. 2012). At BC4, we observed that *D1*^*+*^*Q1*^*+*^ and *D1*^*−*^*Q1*^*−*^ flies did not differ in eye area (*t* = 0.201, df = 46, NS, Fig. 3C), but that *D1*^*+*^*Q1*^*+*^ flies exhibited significantly narrower FWs (*t* = 3.32, df = 46, *P* < 0.01, Fig. 3D). Therefore, our introgression strategy verified the results of our QTL mapping, and directly demonstrates that variation in eye area and FW is genetically separable.

### Variation in overall head shape localizes to the X chromosome

**Figure 4:**
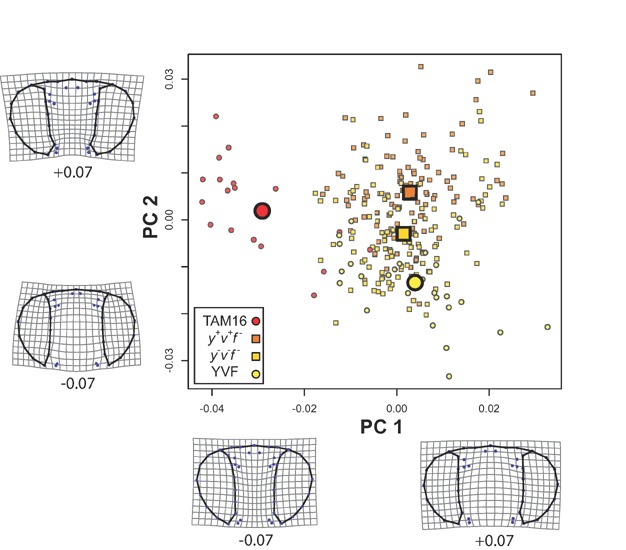
Head shape differences between X introgression lines and parental strains. Principal component (PC) scores are shown for male flies that are hemizygous for the TAM 16 allele (*y^+^v^+^f^−^*; dark orange squares) or the YVF allele (*y^−^v^−^f^−^*; light orange squares) of the EA^TL^ QTL region on the X chromosome at backcross generation 7, as well as the for the parental strains TAM 16 (red circles) and YVF (yellow circles). Small symbols indicate individual flies and large icons group averages. PCs 1 and 2 account for 37% and 28% of the variation, respectively, and deformation grids represent the deviations from the mean shape to ±0.07 on both PC axes, to visualize morphological differences along those axes.

We previously showed that changes in eye size and FW affected the proportions and shape of the head capsule between *D. mauritiana* and *D. simulans* (Posnien et al. 2012). Therefore, we assessed the effect of the X introgression on overall head shape compared to the parental lines using GM. We found two principal components (PCs 1 and 2) that together accounted for 66% of the variation in the dataset (Fig. 4). PC1 clearly separates the parental lines and largely accounts for variation in the relative proportions of face cuticle (Fig. 4). PC2 mostly reflects variation in eye shape and size, with individuals with higher PC2 scores exhibiting a relative enlargement of eye including an expansion of the dorsal region (Fig. 4). Males carrying the introgressed region from *D. mauritiana* (*y*^*+*^*v*^*+*^*f*^*−*^) are different from *D. mauritiana* males for PC1, but not for PC2. Conversely, the males carrying the introgression are not different from *D. simulans* males for PC1 but differ from both recombinant males from the same lines that have lost the introgression (*y*^*−*^*v*^*−*^*f*^−^) and YVF males for PC2. Hence, males carrying the X introgression from *D. mauritiana* have a similar face shape to *D. simulans* but are more like *D. mauritiana* for eye shape, and this is reflected in the overall proportions of these tissues in the head capsule as described by the landmarks we applied.

### The QTL on the X chromosome affects ommatidia size

**Figure 5:**
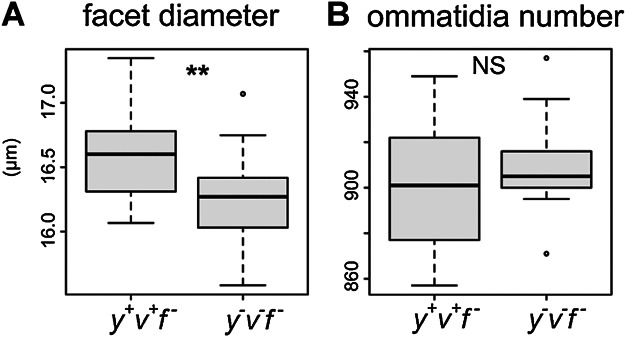
Differences in facet diameter and number in X introgression lines. (A) Boxplot comparing facet diameter, measured as the average of seven facets at the center of the eye, in X introgression males hemizygous for either the TAM16 (*y^+^v^+^f^−^*; *n* = 20, 16.59 ± 0.07 µm) or the YVF (*y^−^v^−^f^−^*, *n* = 20, 16.26 ± 0.06 µm) EA^TL^ QTL region on the X chromosome. (B). Boxplots comparing ommatidia number per eye (*y^+^v^+^f* = 898 ± 6, *y^−^v^−^f^−^* = 908 ± 4) from the same individuals measured in (A).

Differences in eye size between *D. mauritiana* and *D. simulans* are largely the result of differences in ommatidia size, with an average facet diameter difference of approximately 1 µm (Posnien et al. 2012). We therefore measured facet diameter and ommatidia number in our X introgression lines. We found that males carrying the X introgression from *D. mauritiana* (*y*^*+*^*v*^*+*^*f*^*−*^) had significantly larger facets (*t* = 3.01, df = 38, *P* < 0.01, Fig. 5A) compared to their recombinant siblings that had lost the introgressed region (*y*^*−*^*v*^*−*^*f*^*−*^), with an average difference in facet diameter of approximately 0.33 µm. However, ommatidia number did not vary significantly between these flies (*t* = −1.40, df = 38, NS, Fig. 5B). These results indicate that the X introgression from *D. mauritiana* contains allelic variants that are responsible for increased ommatidia size that contribute to the larger eye size of this species compared to *D. simulans*.

### Differences in the relative size of the face and eye primordia are already established during larval stages

**Figure 6:**
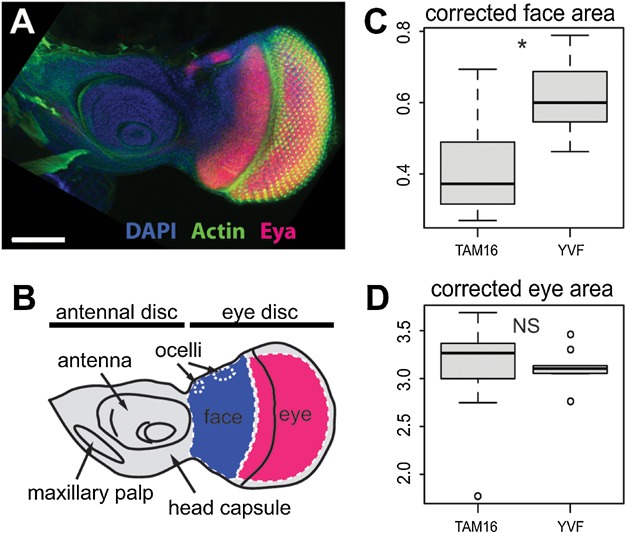
Differences in the relative size of the face primordia are already established during larval stages. (A) Immunostaining of eye-antenna imaginal disc with anti-Eya (magenta). Nuclei are marked with DAPI (blue) and cellular membranes are shown in green (Phalloidin). (B) Scheme showing the compartments of the eye-antennal disc that will give rise to the different structures of the adult head. Colors indicated the regions that were defined using the markers shown in (A), and of which the surface areas were measured in this study: face (blue) and eye (magenta). (C–D) Boxplots showing the relative areas of eyes (C) and faces (D) from TAM16 (*n* = 9, corrected face area = 0.426 ± 0.054, corrected eye area = 3.075 ± 0.187) and YVF (*n* = 10, 0.616 ± 0.030, 3.118 ± 0.057) imaginal discs.

To investigate the developmental basis of the differences in adult head morphology, we studied the relative proportions of the eye and face primordia in the eye-antennal discs of third instar larvae of *D. simulans* and *D. mauritiana*. To do this we measured the expression of Eyes absent (Eya), which defines the eye primordium within the eye-antenna disc (Bonini et al. 1993; Bonini et al. 1997; Chen et al. 1997; Pignoni et al. 1997), in relation to the area of the primordium of the face at 120 h after egg laying (i.e., when the morphogenic furrow has almost completed its progression). Note that we approximated the face primordium as the tissue between Eya expression and the morphological border of the antennal primordium (Fig. 6A and B). We found that the size of the eye primordia, normalized by the area of the whole disc, was not significantly different between the species (*t* = 0.225, df = 9.46, NS, Fig. 6C). However, the face primordia of *D. simulans* were significantly larger than those of *D. mauritiana* (*t* = 3.076, df = 12.79, *P* < 0.01). This shows that the differences between *D. simulans* and *D. mauritiana* adult faces are already established during larval development but that the difference in the size of the retinal field develops later (Fig. 6D).

## DISCUSSION

### Variation in eye and face size has a polygenic basis and includes regions of large effect

There is considerable variation in the size of compound eyes and other head capsule tissues among insects including *Drosophila*. Therefore, to study the evolution of changes in head capsule tissues, we investigated the genetic basis of differences in eye size and FW between *D. mauritiana* and *D. simulans* using a combination of quantitative genetic, introgression, and developmental approaches.

We found that variation in both eye size and FW is polygenic and includes some regions that have a large effect on either trait. For example, the QTL on the X chromosome explains approximately 25% and 44% of the raw and corrected parental differences in eye size, respectively. This is consistent with the effect size of the X introgression: 25% and 22% for raw and corrected eye area, respectively, although this shows that the QTL effect may be overestimated when the eye size is corrected for body size. Our QTL analyses also revealed regions of large effect for FW, on chromosomes 2L and 3L, with effects (in opposite directions) of more than 100% of the difference between the parental lines (Table 2). Indeed, introgression of a large region of 3L from *D. mauritiana* into *D. simulans* resulted in a narrower face which, when corrected for body size, corresponds to more than double the difference between the parental lines. Interestingly, such transgressive phenotypes have been reported in hybrids of ecologically and reproductively isolated parental species (Rieseberg et al. 1999; Bell and Travis 2005).

Given that we have identified several QTL of large effect for eye and FW, our findings may support models of phenotypic evolution involving fixation of changes of large effect followed by further multiple changes of smaller effect (with the caveat we do not know if the loci of large effect identified in our study were fixed first) (reviewed in Orr 2005). Moreover, although our tests support single QTL models within the regions of large effect we detected for these two traits, care must be taken in interpreting the number and effect size of QTL because they can encompass multiple genes, and each of these could have accumulated many individual mutations (Frankel et al. 2011). Indeed, given the size of our mapping population, QTL of small effect may have been missed in our analysis. Further high-resolution mapping is required to identify the genes and nucleotide changes underlying these QTL and then test the effects of these changes individually and in combination with other regions.

### Changes in eye size and FW are caused by variation at different loci

Intra and interspecific comparisons among *Drosophila* species have revealed that there is a consistent negative correlation between eye size and FW (Cowley and Atchley 1990; Norry et al. 2000; Posnien et al. 2012). Therefore, there may be physical constraints on the overall size of the head capsule. Furthermore, it is also possible that there are genetic constraints given the juxtaposition of these tissues in the developing eye-antennal disc, and so variation in the same pathway or even the same gene could make one tissue larger and the other smaller. In this scenario, the overlap of loci controlling variation in each trait is largely determined by the level of their developmental integration (Cheverud 1996; Wagner 1996). However, there is no evidence for any functional integration between eye size and FW, and it is clear from both our QTL mapping and introgression-based approach that different loci underlie variation in eye size and FW, respectively. The chromosome 3 introgression from *D. mauritiana* into *D. simulans* resulted in flies with narrower faces, but no significant difference in eye size. Reciprocally, the chromosome X introgression (in the same direction) gave rise to flies with larger eyes but no significant difference in FW, which is evident from both direct measurements and the PCs from our GM analysis. While we cannot exclude the possibility of variation in further loci of smaller effect regulating the size of both tissues, at least the largest effects appear to be genetically independent. Furthermore, the results of our mapping experiments are consistent with our finding that the difference in FW is apparent earlier in development than the reciprocal changes in eye size. This makes sense in a developmental context because the difference in eye size between these species is caused by an enlargement of ommatidia in *D. mauritiana* (Posnien et al. 2012), which is determined later in development than the determination of the presumptive eye field by mutually antagonistic Wg and Dpp signaling (Dominguez and Casares 2005).

Thus, our genetic and developmental findings from studying natural variation are consistent with the effects of known mutations that perturb head capsule development, and together these results suggest that eye size and FW have the potential to evolve independently in *Drosophila*. Indeed changes in the proportions of tissues are observed in the head capsule morphology of other insects. For example, while the males of some blowfly species have reduced face cuticle compared to females, the eye size of the sexes is the same (Sukontason et al. 2008). Stalk eyed flies also exhibit large differences in their head cuticle that do not affect the eye size (Wilkinson and Dodson 1997). It is possible that the negative correlation between eye and FW observed in *Drosophila* could be explained by evolutionary forces acting to enlarge one of these structures and the other tissue is then subject to a compensatory reduction in size.

### Functional significance of changes in eye size and/or FW?

Our results reported here, and from our previous work, show that the eyes of *D. mauritiana* are larger than those of *D. simulans* and, in particular, that they are enlarged in the dorsal region. This is consistent with relatively higher expression of *rhodopsin 3* in *D. mauritiana* than *D. simulans* (Posnien et al. 2012). Therefore, the gain of larger ommatidia, likely conferring greater light sensitivity to the specialized dorsal region of the eye (Land 1997), may have facilitated the evolution of the vision of *D. mauritiana*. A precedent for this is found in the sexual dimorphism in ommatidia subtypes in *Musca domestica* (Franceschini et al. 1981; Land and Eckert 1985). However, it remains possible that sexual selection may be responsible for the differences in FW, and therefore the distance between the eyes, of *D. mauritiana* and *D. simulans*, as has been described for stalked-eyed flies (reviewed in Emlen and Nijhout 2000). Further experiments to test the behavioral consequences of variation in eye size and FW caused by the genes in the regions identified in this study is required to test these hypotheses.

## Supporting Information

Figure S1 Phenotypic measurements used in the study. Frontal image of a *Drosophila* head showing: (A) the linear measurements used to measure eye area (red outlines) and face width (blue arrow); (B) Landmarks used for geometric morphometric analysis. White landmarks are fixed while gray landmarks were used as sliding semi-landmarks. Scale bars = 200 μm.

Figure S2 Crossing schemes used to generate the X and 3L introgression lines. See main text for details.

Figure S3 QTL for Eye Area, Face Width, and Tibia Length. LOD profiles for eye area (EA), face width (FW) and tibia length (TL) based on CIM analysis. Maps for EA and FW are based on raw measurements without taking body size into account. Dashed line represents an arbitrary significance threshold of LOD = 3. Ticks on the x-axis represent genotyped markers spaced in cM across chromosomes 2, 3 and X.

Figure S4 Standard Interval Mapping QTL affecting EA^TL^ and FW^TL^. LOD profiles for (A) eye area (EA) and (B) face width (FW) with tibia length (TL) as a covariate from two rounds of single QTL scans. Black curves represent the first scan where the highest peak on these was used as an additional QTL covariate for a second single QTL scan (gray curve). Dashed lines are a = 0.01 significance thresholds (EA^TL^ = 2.69, FW^TL^ = 2.67) based on 1000 permutations of the dataset.

Table S1 Excel file of all markers used in this work enclosed separately.
