# Supplementary figures and images for: Genetic and developmental analysis of differences in eye and face morphology between Drosophila simulans and Drosophila mauritiana
Source: Evol Dev. 2013 May 16;15(4):257–67. doi: 10.1111/ede.12027 (PMC3799016; doi:10.1111/ede.12027)

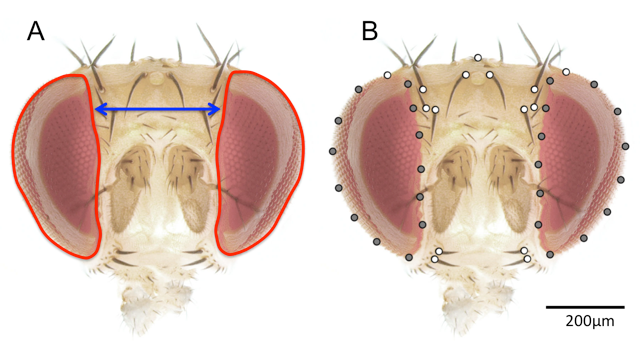

Supplement: Figure S1 — Phenotypic measurements used in the study. Frontal image of a Drosophila head showing: (A) the linear measurements used to measure eye area (red outlines) and face width (blue arrow); (B) Landmarks used for geometric morphometric analysis. White landmarks are fixed while gray landmarks were used as sliding semi-landmarks. Scale bars = 200 μm. [file ede0015-0257-sd1.tif]

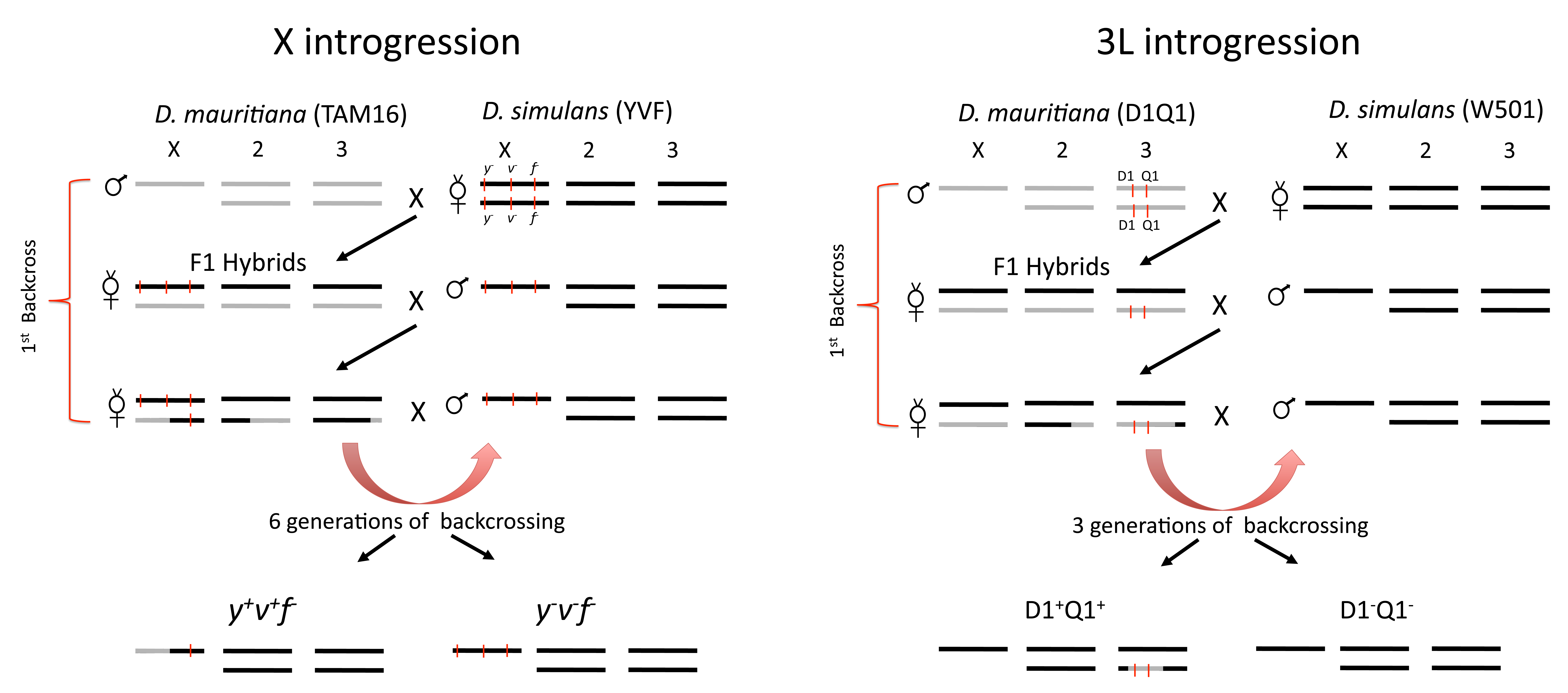

Supplement: Figure S2 — Crossing schemes used to generate the X and 3L introgression lines. See main text for details. [file ede0015-0257-sd2.tif]

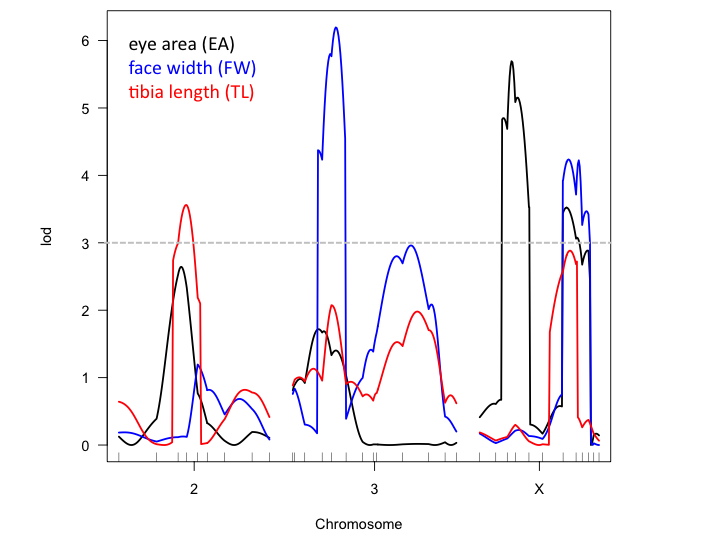

Supplement: Figure S3 — QTL for Eye Area, Face Width, and Tibia Length. LOD profiles for eye area (EA), face width (FW) and tibia length (TL) based on CIM analysis. Maps for EA and FW are based on raw measurements without taking body size into account. Dashed line represents an arbitrary significance threshold of LOD = 3. Ticks on the x-axis represent genotyped markers spaced in cM across chromosomes 2, 3 and X. [file ede0015-0257-sd3.tif]

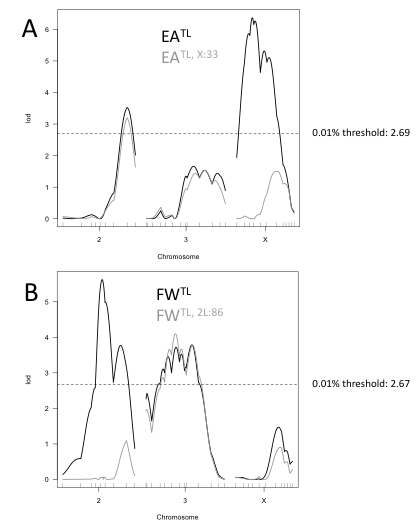

Supplement: Figure S4 — Standard Interval Mapping QTL affecting EATL and FWTL. LOD profiles for (A) eye area (EA) and (B) face width (FW) with tibia length (TL) as a covariate from two rounds of single QTL scans. Black curves represent the first scan where the highest peak on these was used as an additional QTL covariate for a second single QTL scan (gray curve). Dashed lines are a = 0.01 significance thresholds (EATL = 2.69, FWTL = 2.67) based on 1000 permutations of the dataset. [file ede0015-0257-sd4.tif]
